# Prognostic factors for mortality in bullous pemphigoid: A systematic review and meta-analysis

**DOI:** 10.1371/journal.pone.0264705

**Published:** 2022-04-15

**Authors:** Xianxia Chen, Yaqiang Zhang, Zhicheng Luo, Yujuan Wu, Taoxiang Niu, Jiayuan Zheng, Yuanyuan Xie

**Affiliations:** 1 Department of Dermatology, Lanzhou University Second Hospital, Gansu, China; 2 The 940th Hosptial of Joint Logistics Support force of Chinese People’s Liberation Army, Gansu, China; Northwestern University Feinberg School of Medicine Galter Health Sciences Library, UNITED STATES

## Abstract

**Objective:**

To systematically evaluate the prognostic factors for mortality in bullous pemphigoid.

**Methods:**

PubMed, Embase, Cochrane Library, China National Knowledge Infrastructure, China Biology Medicine disc and Wanfang Database were searched to collect literature on the prognostic factors for mortality in bullous pemphigoid. The quality of studies was assessed by Newcastle–Ottawa Quality Assessment Scale. Two researchers extracted relevant data and scored study quality independently. The hazard ratio (HR) was calculated using the random effects model. Study heterogeneity was assessed using both Cochran’s Q test and I^2^ statistics. The causes of heterogeneity were assessed by subgroup analysis and/ or sensitivity analysis when heterogeneity was significant. When ten or more studies were included as outcome indicators, publication bias was evaluated by funnel plot and Egger’s test.

**Results:**

Out of a total of 1,546 articles retrieved, 15 studies involving 2,435 patients were included. The meta-analysis showed that the mortality of patients with bullous pemphigoid increased with positive bullous pemphigoid 180 antibody (HR = 1.85, 95%CI: 1.25~2.75, *P* = 0.002); concomitant dementia (HR = 2.26, 95%CI: 1.43~3.59, *P*<0.001); stroke (HR = 2.09, 95% CI: 1.23–3.55, *P* = 0.007); heart disease (HR = 1.96, 95% CI: 1.41–2.73, *P*<0.001) and diabetes mellitus (HR = 2.39, 95% CI: 1.55–3.69, *P*<0.001). Sex, positive indirect immunofluorescence and hypertension were not associated with prognosis.

**Conclusion:**

Positive bullous pemphigoid 180 antibody, dementia, stroke, heart disease and diabetes mellitus were the prognostic factors for mortality in bullous pemphigoid.

## Introduction

Bullous pemphigoid is the most common subepidermal autoimmune bullous disease. Its occurrence is related with the production of autoantibodies by hemidesmosome-anchored proteins bullous pemphigoid 180 and bullous pemphigoid 230, which leads to the separation of the epidermis and dermis with antigen and antibody combination. Bullous pemphigoid is characterised by tension blisters on the background of normal or erythematous skin and negative Nikolsky sign; it is often accompanied by severe itching and a few have mucosal damage [1]. This disease is prevalent in the elderly, particularly with a high incidence in those over 70 years old. Researches showed that the average annual incidence rate was 2.5 to 42.8 cases per million people worldwide [2–5]. In the past 20 years, the incidence rate of bullous pemphigoid has increased by 1.9 to 4.3 times because of the ageing population, presence of various complications, drugs that may cause diseases and improvement of clinical diagnosis and laboratory technology [6,7]. Because bullous pemphigoid is a chronic and recurrent severe skin disease, most patients die of body consumption failure caused by long-term illness, complications and multiple organ failure caused by long-term use of glucocorticoids. According to global data statistics, the one-year mortality rate of bullous pemphigoid was 23.5% [8].

Because of the high incidence and mortality rates, most studies have focused on the prognostic factors for mortality in bullous pemphigoid, but the results varied. The earliest studies showed that the presence of bullous pemphigoid 180 antibody was the first confirmed prognostic factor for mortality in bullous pemphigoid [9]. Subsequently, advanced age [10–23], sex [17–19,21–23], neurologic diseases [10,11,13,16,19,21,23] and heart disease [12–21] were found to affect the prognosis of bullous pemphigoid. However, the prognostic effects of closely related factors, such as the general condition of patients [14,16,17,23] and disease severity [10,13,21,23], were controversial. Since most of these studies on prognostic factors were retrospective, had a relatively small sample size and uneven quality, the results obtained had certain limitations. Consequently, we conducted a systematic review and meta-analysis to comprehensively evaluate the evidence-based factors that influence the prognosis of bullous pemphigoid.

## Methods

### Search strategy

We searched Pubmed, Embase, Cochrane Library, China National Knowledge Infrastructure, China Biology Medicine disc and Wanfang Database using the keywords ‘bullous pemphigoid’, ‘pemphigoid’, ‘prognosis’ and ‘mortality’. Meanwhile, the references of the included literature were manually searched. The search time limit was from the time of establishment of each database to April 2020.

### Inclusion and exclusion criteria

The inclusion criteria were (1) studies in which bullous pemphigoid was diagnosed by consistent clinical features and histopathological evidence of subepidermal blister formation and/or immunopathology (including direct immunofluorescence / indirect immunofluorescence / bullous pemphigoid 180 antigen detected by enzyme-linked immunosorbent assay or by Western blot analysis), (2) studies that evaluated the prognostic factors for mortality in bullous pemphigoid and (3) observational cohort and case-control studies. The exclusion criteria were (1) case reports, reviews, meetings, abstracts, letters and meta-analysis; (2) studies with incomplete data or unavailable full text or effective literature and (3) studies with outcome events of remission or recurrence, not mortality.

### Study selection and data extraction

Literature was screened based on the information available in the title and abstract. The full text of the studies were reviewed for eligibility in the meta-analysis. The literature screening was performed independently by two researchers, and disagreements were resolved through discussion or by the third researcher. All data were extracted independently by two researchers and included authors, year of publication, countries or regions where the studies were conducted, research type, sample size, follow-up time, endpoint indicators, outcome evaluation indicators and results. If a study reported one-year mortality and overall mortality, one-year mortality was extracted for analysis. The other researchers were responsible for checking the extracted data to ensure authenticity and accuracy.

### Quality assessment

The quality of the included literature was scored independently by two researchers, according to the Newcastle–Ottawa Quality Assessment Scale (NOS); inconsistent scores were judged by the third researcher. The full score of NOS was nine stars and included the quality of selection, comparability and outcome of the study participants. High quality research was defined as seven or more stars [24].

### Statistical analysis

The statistical analysis was performed by the Review Manager 5.4 software (Copenhagen: the Nordic Cochrane Centre, the Cochrane Collaboration, 2020). The hazard ratio (HR) with 95% confidence interval (CI) obtained by multivariable analysis was extracted for the effect variables. For studies in which HR with 95% CI was not given directly, the original data were used for calculation. In few situations where HR could not be calculated, the relative risk ratio (RR) with 95% CI was obtained instead [25]. The results were combined for analysis if three or more studies found a factor that was associated with prognosis, the random effects models of DerSimonian and Laird methods were used for data consolidation [26]. Statistical significance was considered when the P-value was <0.05. Study heterogeneity was assessed using both Cochran’s Q test and I^2^ statistics. Statistical significance of heterogeneity was set at *P*<0.1, and the magnitude was interpreted as no (0%≤ I^2^<25%), mild (25%≤ I^2^<50%), moderate (50%≤ I^2^<75%) or substantial (I^2^≥75%) [27]. The causes of heterogeneity were assessed by subgroup analysis and/ or sensitivity analysis when heterogeneity was significant (*P*<0.1 and I^2^≥50%). If analysis of the causes of heterogeneity was impossible, only qualitative and systematic evaluation was performed. When ten or more articles were included as outcome indicators, publication bias was evaluated by funnel plot and Egger’s test using Stata 16.0 (StataCorp, College Station, TX, USA); *P*<0.05 was considered publication bias. Adjustment by the trim and fill method was performed if there was doubt about publication bias.

## Results

### Included studies

**Fig 1:**
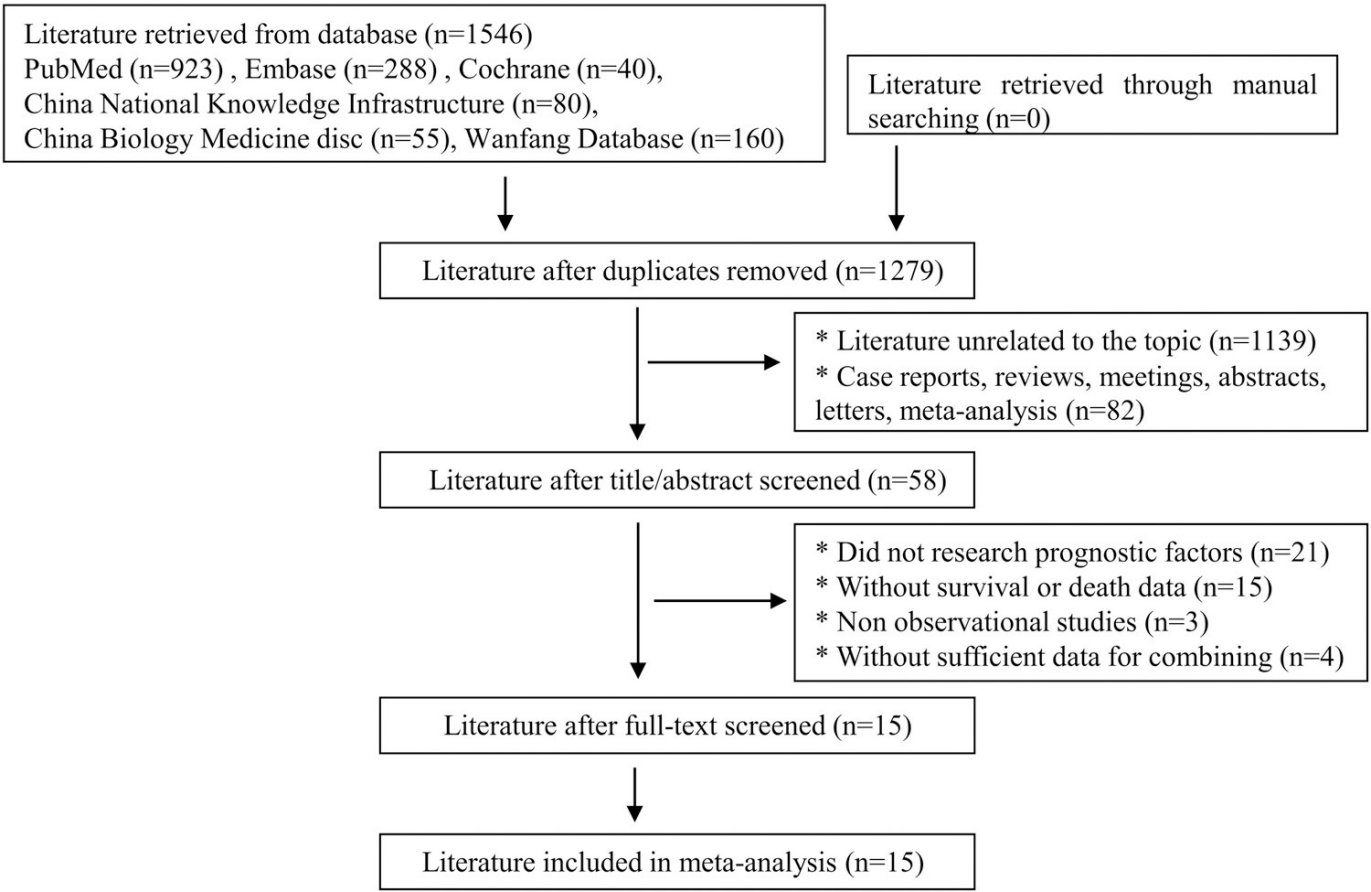
The flowchart of literature screening.

A total of 1,546 articles were retrieved. According to the inclusion and exclusion criteria, 15 articles involving 2,435 patients were selected. The process and results of the literature screening are shown in Fig 1. Of the 15 articles, 2 were prospective studies and 13 were retrospective studies. The basic characteristics of the included studies are shown in Table 1. According to the NOS scale, all studies were considered to be of high quality (Table 2).

**Table 1:** The basic characteristics of included studies.

Inclusion studies	Country	Sample size	Research type	Endpoint indicators	Outcome evaluation indicators
Monshi 2020 [10]	Austria	100	Retrospective	1-year mortality	(1) (3) (6) (9) (20)
Rozenblat 2019 [11]	Israeli	87	Retrospective	1-year mortality	(1) (9) (19)
Jeon 2018 [12]	Korea	103	Retrospective	Overall mortality	(1) (14) (16)
Kalinska 2017 [13]	Poland	205	Retrospective	1-year/Overall mortality	(1) (3) (9) (11) (12) (13) (14) (20)
Lee 2014 [14]	Korea	168	Retrospective	1-year/Overall mortality	(1) (5) (10) (14) (15)
Gual 2014 [15]	Spain	101	Retrospective	1-year mortality	(1) (14) (20)
Cai 2014 [16]	Singapore	359	Retrospective	Overall mortality	(1) (4) (5) (9) (10) (12) (13) (14) (16) (17)
Zhang 2013 [17]	China	94	Retrospective	1-year mortality	(1) (2) (5) (10) (13) (14) (15)
Li 2013 [18]	China	140	Retrospective	Overall mortality	(1) (2) (7) (8) (14) (15)
Cortés 2012 [19]	Switzerland	60	Retrospective	1-year/Overall mortality	(1) (2) (6) (7) (9) (13) (14) (15)
Cortés 2011 [20]	Switzerland	115	Prospective	Overall mortality	(1) (14)
Parker 2008 [21]	USA	223	Retrospective	1-year mortality	(1) (2) (3) (9) (10) (13) (14) (15)
Rzany 2002 [22]	Germany	369	Retrospective	1-year mortality	(1) (2) (8)
Roujeau 1998 [23]	France	217	Retrospective	6 month mortality	(1) (2) (3) (5) (9) (18)
Bernard 1997 [9]	France	94	Prospective	1-year mortality	(6) (7)

Note: (1)Advanced age (2)Gender (3)Disease severity (4)Mucosal lesion (5)Poor general condition (6)Positive bullous pemphigoid 180 antibody (7)Positive indirect immunofluorescence (8)Low serum albumin level (9)Concomitant dementia (10)Concomitant stroke (11)Concomitant epilepsy (12)Concomitant Parkinson disease (13)Concomitant hypertension (14)Concomitant heart disease (15)Concomitant diabetes mellitus (16)Concomitant kidney disease (17)Concomitant malignancy (18)Corticosteroid treatment alone (19)Statins intake(20)Hospitalization(days).

**Table 2:** The quality assessment of included studies.

Inclusion studies	Selection	Comparability	Outcome	Total score
Monshi 2020 [10]	4	2	2	8
Rozenblat 2019 [11]	4	2	2	8
Jeon 2018 [12]	4	2	2	8
Kalinska 2017 [13]	4	2	2	8
Lee 2014 [14]	4	2	2	8
Gual 2014 [15]	4	2	3	9
Cai 2014 [16]	4	2	2	8
Zhang 2013 [17]	4	2	3	8
Li 2013 [18]	4	2	2	8
Cortés 2012 [19]	4	2	2	8
Cortés 2011 [20]	4	2	3	9
Parker 2008 [21]	4	1	2	7
Rzany 2002 [22]	4	2	3	9
Roujeau 1998 [23]	4	2	2	8
Bernard 1997 [9]	4	0	3	7

### Outcomes of meta-analysis

#### 1. Age

**Fig 2:**
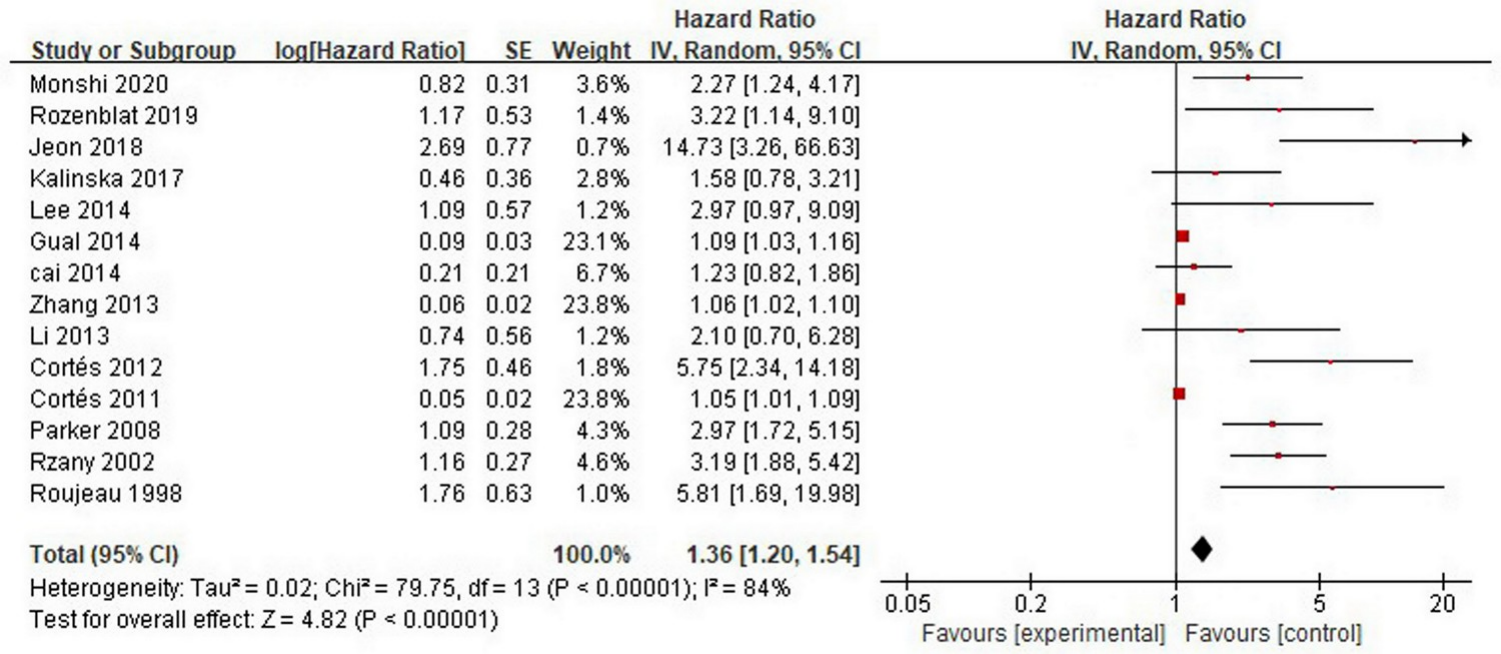
Forest plot of the association between advanced age and bullous pemphigoid mortality.

As shown in Fig 2, 14 studies [10–23] analysed the association between advanced age and mortality in patients with bullous pemphigoid. In most studies, the median or average age at diagnosis was used as the cut-off value for advanced age, which ranged from 67 to 85 years among the studies. Meta-analysis showed that the overall pooled HR of advanced age was 1.36 (95%CI: 1.20–1.54, *P*<0.001), and the results were statistically significant. Substantial heterogeneity was found among the studies (*P*< 0.001, I^2^ = 84%).

**Fig 3:**
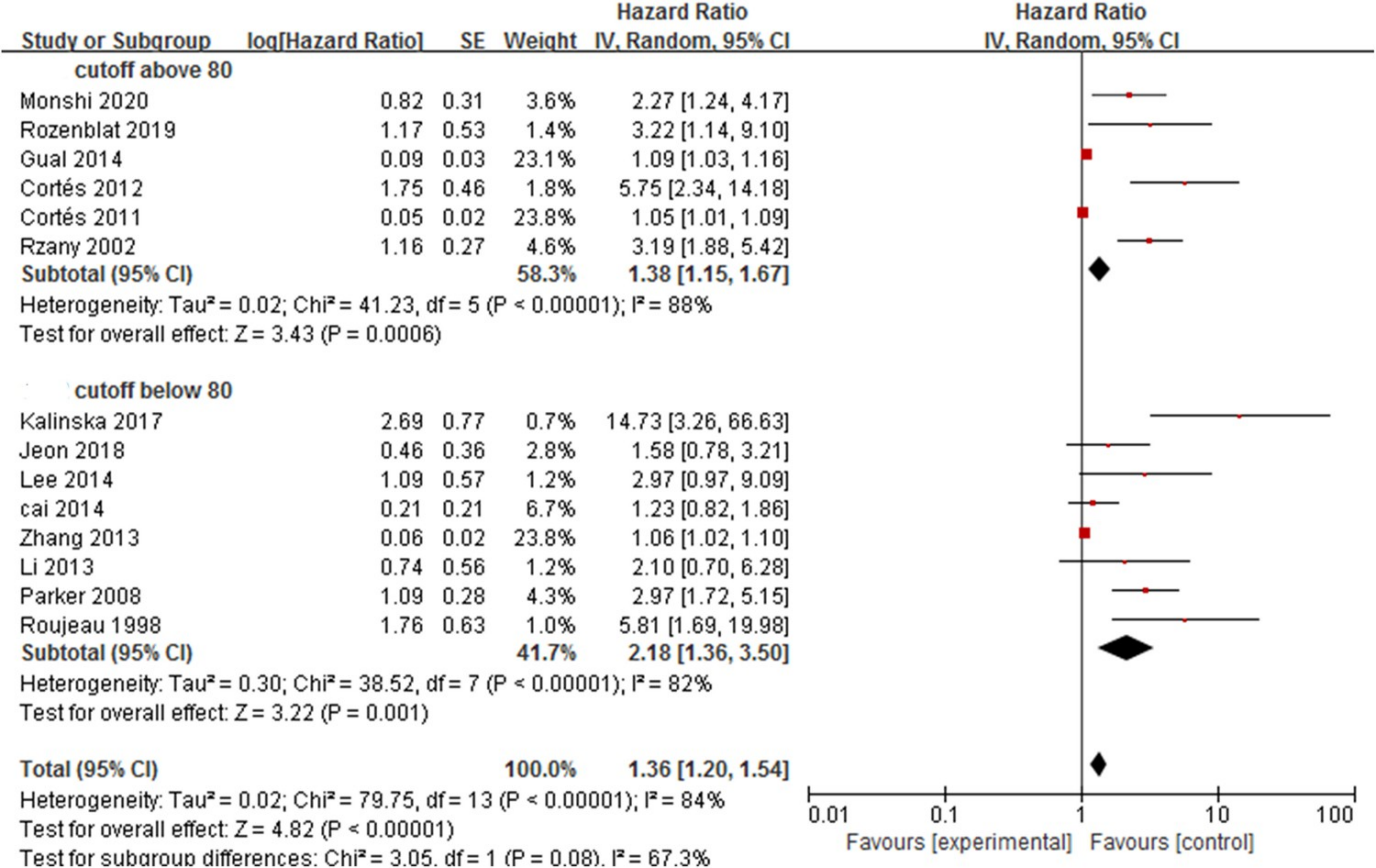
Forest plot of the subgroup analysis of the association between advanced age and bullous pemphigoid mortality.

Subgroup analysis for advanced age (Fig 3) showed that the pooled HR of a cut-off age of ≥80 years was 1.38 (95%CI: 1.15–1.67, *P*<0.001) and that of <80 years was 2.18 (95%CI: 1.36–1.54, *P* = 0.001); both results were statistically significant. The heterogeneity test of the two subgroups showed the following: age ≥80 years (*P*< 0.001, I^2^ = 88%) and age <80 years (*P*<0.001, I^2^ = 82%).

The sensitivity analysis was performed on the studies. There was no change in the effect and similar heterogeneity after the exclusion of each study one by one. The results were still statistically significant (HR = 1.23, 95%CI: 1.10–1.38, *P*<0.001), and substantial heterogeneity was found (*P*<0.001, I^2^ = 80%) after excluding the studies [22,23] that provided RR. The result showed that HR = 1.44(95%CI: 1.21–1.72, *P*<0.001), and the heterogeneity was substantial (*P*<0.001, I^2^ = 84%) after excluding the studies in which the endpoint indicator was not one-year mortality [12,16,18–20,23].

**Fig 4:**
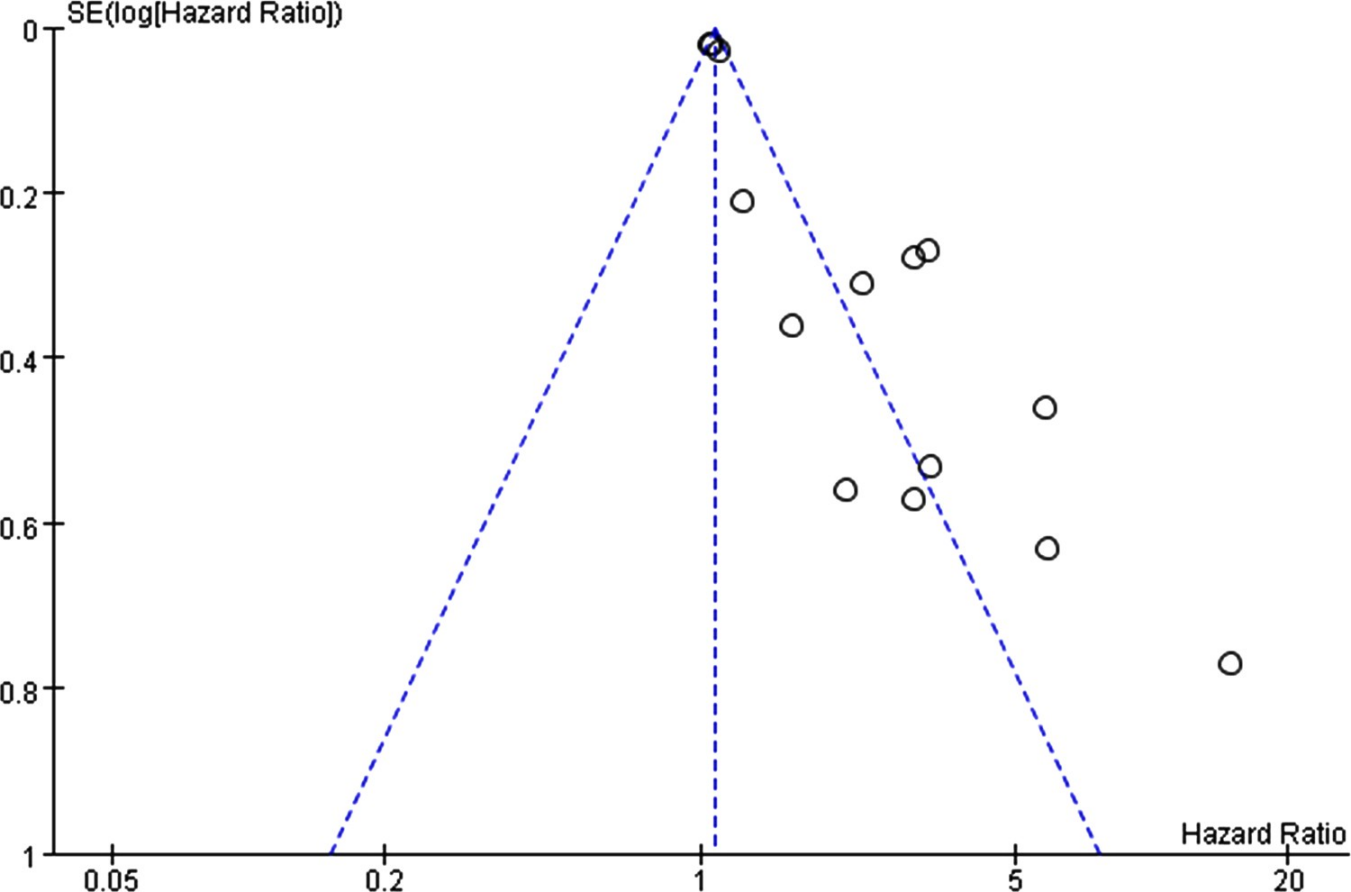
Funnel plot of the association between advanced age and bullous pemphigoid mortality.

**Fig 5:**
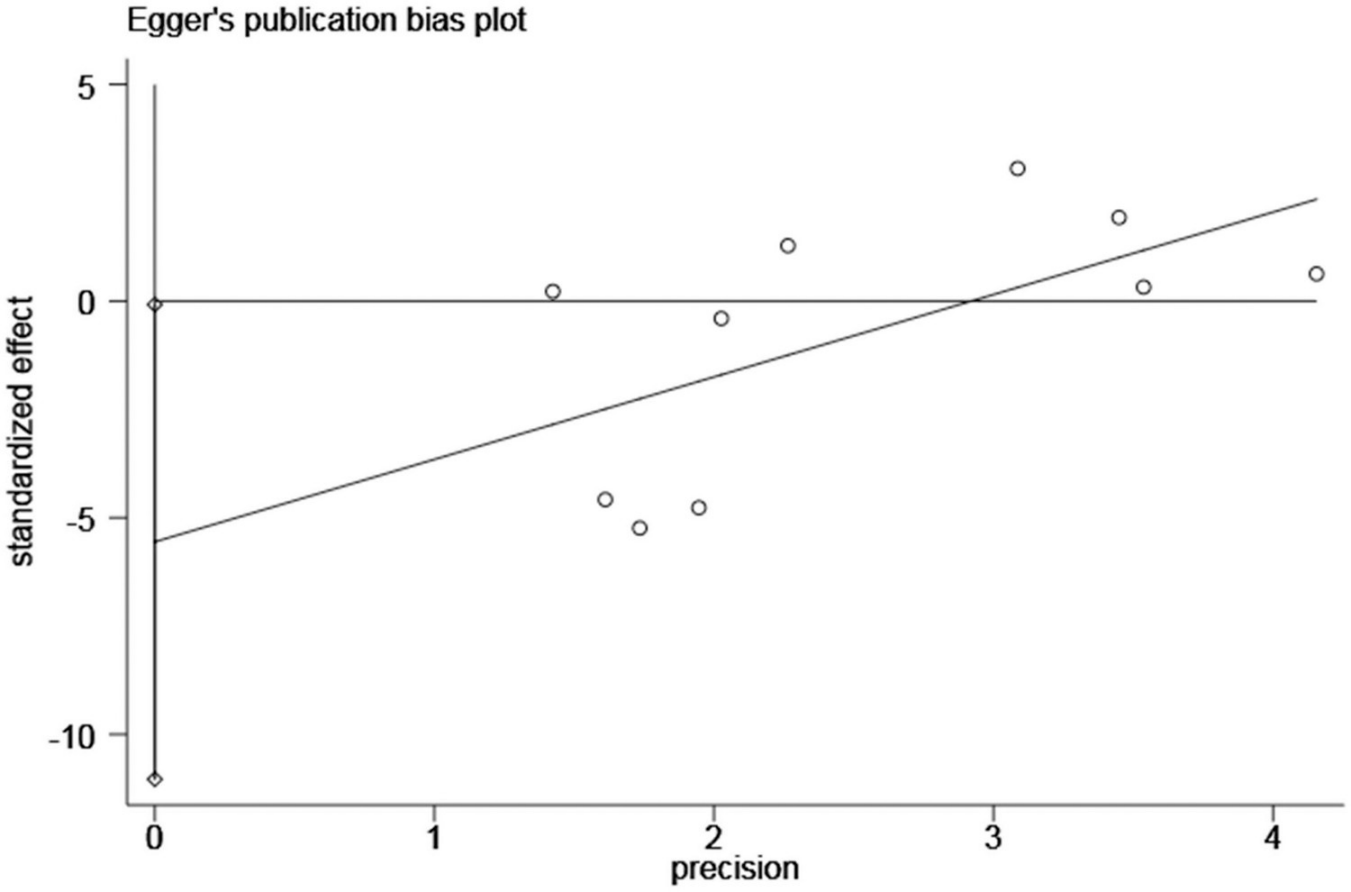
Egger’s publication bias plot of the association between advanced age and bullous pemphigoid mortality.

**Fig 6:**
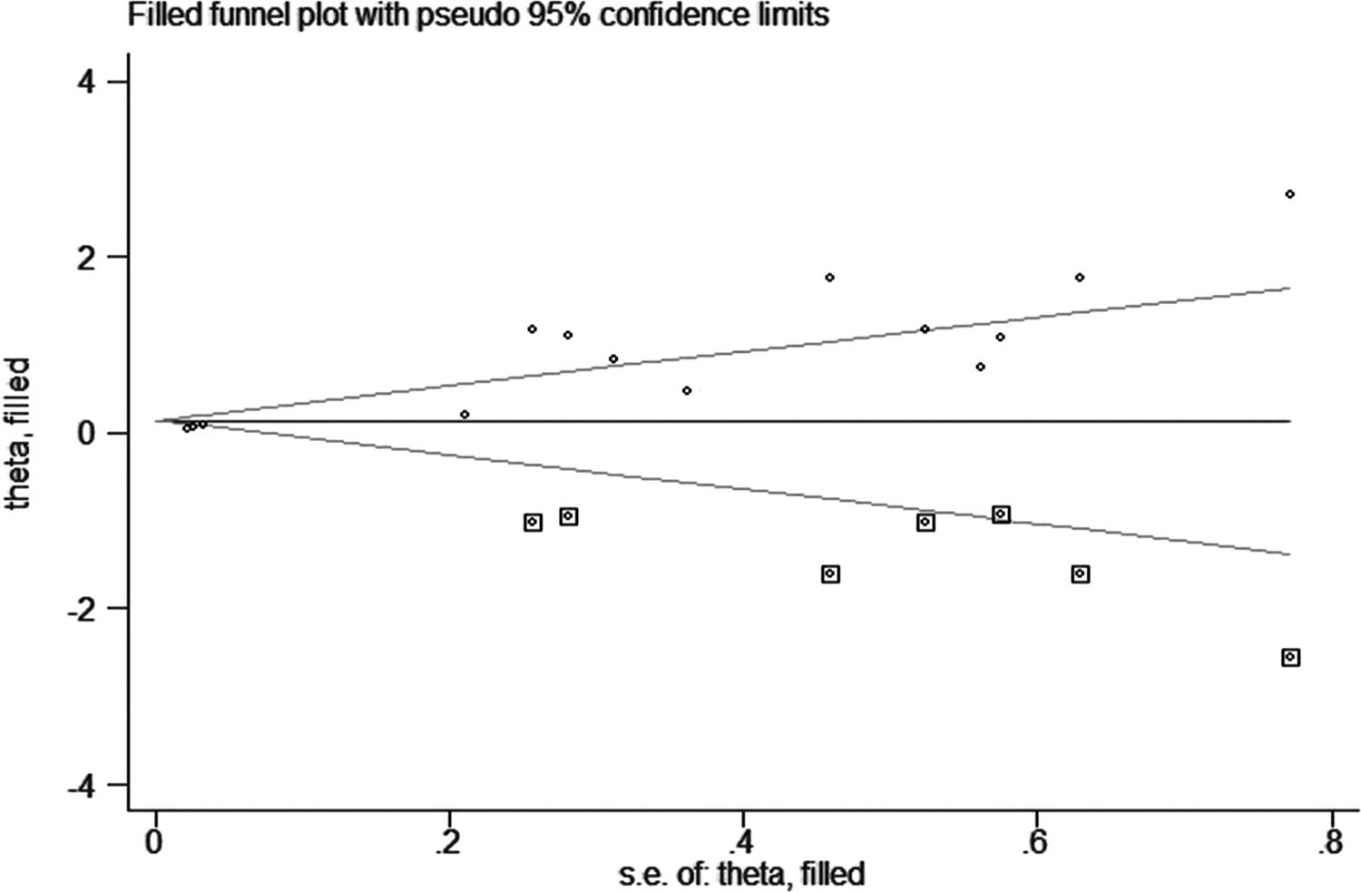
Trim and fill plot of publication bias on the association between advanced age and bullous pemphigoid mortality.

The shape of the funnel plot was asymmetric and deviated from the dotted line (Fig 4), and Egger’s test was statistically significant (*P*<0.05) (Fig 5), indicating the presence of publication bias, which was adjusted by the trim and fill method. After adding seven studies, the funnel plot was symmetrical and concentrated (Fig 6), but the result was not statistically significant (HR = 1.15, 95%CI: 0.98–1.35, *P* = 0.082).

#### 2. Sex

**Fig 7:**
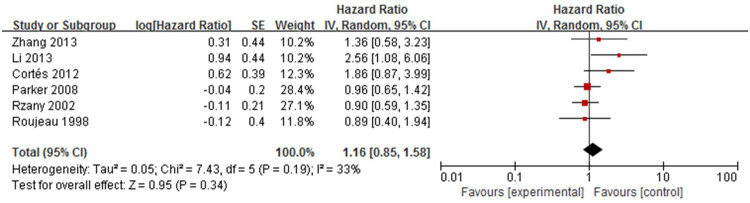
Forest plot of the association between sex and bullous pemphigoid mortality.

Six studies [17–19,21–23] analysed the association between sex and mortality in patients with bullous pemphigoid (Fig 7). The overall pooled HR of sex was 1.16(95%CI: 0.85~1.58, *P* = 0.34), and the results were not statistically significant. Mild heterogeneity was found across the studies (*P* = 0.19, I^2^ = 33%).

#### 3. Disease severity

**Fig 8:**
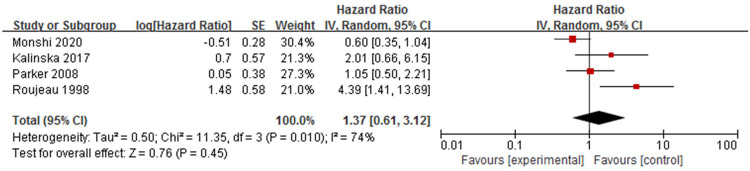
Forest plot of the association between disease severity and bullous pemphigoid mortality.

Four studies [10,13,21,23] analysed the association between disease severity and mortality (Fig 8), but the assessment of disease severity was different among the studies. Monshi [10] assessed the severity by the autoimmune bullous skin disorder intensity score. Kalinska [13] and Parker [21] graded the severity by the percentage of involved body surface area as mild (<10%), moderate (10% to 30%) and severe (>30%). Roujeau [23] divided severity into local or extensive lesions, according to the degree of pruritus, number of blisters and levels of eosinophils and autoantibodies. Meta-analysis showed that the pooled HR of disease severity was not statistically significant (HR = 1.37, 95%CI: 0.61–3.12, *P* = 0.45). There was moderate heterogeneity among the studies (P = 0.01, I^2^ = 74%).

The sensitivity analysis showed an unchanged effect with high heterogeneity after the exclusion of each study one by one. The results showed no statistical significance (HR = 0.95, 95%CI: 0.50~1.79, *P* = 0.87) and moderate heterogeneity (*P* = 0.13, I^2^ = 52%) after excluding the research [23] that provided RR and not one-year mortality. However, an unchanged effect with no heterogeneity was found (*P* = 0.34, I^2^ = 0%) after both studies of Monshi [10] and Roujeau [23] were removed. Therefore, different assessment methods of disease severity might have been the source of high heterogeneity in the entire study.

#### 4. General condition

**Fig 9:**
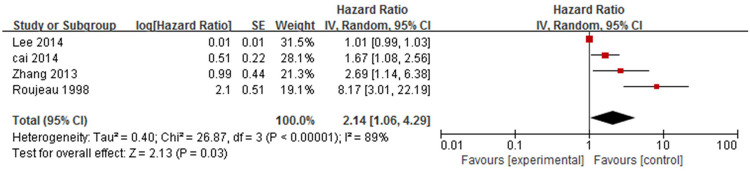
Forest plot of the association between the general condition and bullous pemphigoid mortality.

The general condition of patients was evaluated by the Karnofsky performance status scale (KPS), which ranged from 100 (normal function) to 0 (death) [28]. The association between general condition and mortality in patients with bullous pemphigoid was analysed in four studies [14,16,17,23] (Fig 9), three of which [14,16,23] were assessed by KPS. Meta-analysis showed a combined HR of 2.14 (95%CI: 1.06–4.29, *P* = 0.03), and the results were statistically significant. There was significant heterogeneity among the studies (*P*<0.001, I^2^ = 89%).

The sensitivity analysis showed similar heterogeneity after removing each study one by one. However, the effect was changed and the results were not statistically significant after removing the studies of Cai [16], Zhang [17] and Roujeau [23] one by one, indicating poor stability of the entire study. The result was not statistically significant (HR = 1.46, 95%CI: 0.87–2.47, *P* = 0.15) and substantial heterogeneity was found (*P =* 0.006, I^2^ = 80%) after removing the studies [23] that provided RR. There had no statistical significance (HR = 1.49, 95%CI: 0.58–3.83, *P* = 0.40) and substantial heterogeneity (*P* = 0.03, I^2^ = 80%) after excluding the studies [16,23] that endpoint indicator was not one-year mortality.

#### 5. Positive bullous pemphigoid 180 antibody

**Fig 10:**
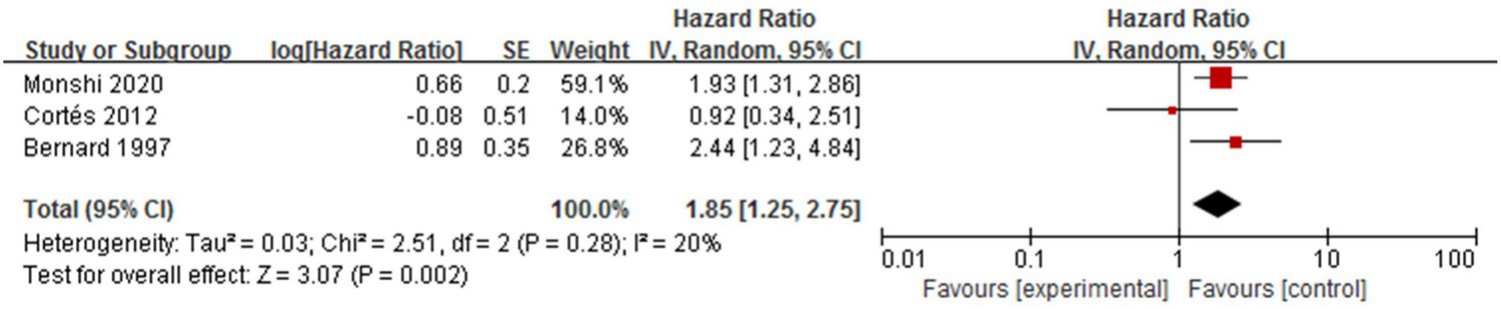
Forest plot of the association between positive bullous pemphigoid 180 antibody and bullous pemphigoid mortality.

Three studies [9,10,19] analysed the association between positive bullous pemphigoid 180 antibody and mortality in patients with bullous pemphigoid (Fig 10). The presence of the bullous pemphigoid 180 antibody was detected by enzyme-linked immunosorbent assay (ELISA) or Western blot. The overall pooled HR was statistically significant (HR = 1.85, 95%CI:1.25–2.75, *P* = 0.002). There was no heterogeneity among the studies (*P* = 0.28, I^2^ = 20%).

#### 6. Positive indirect immunofluorescence (IIF)

**Fig 11:**
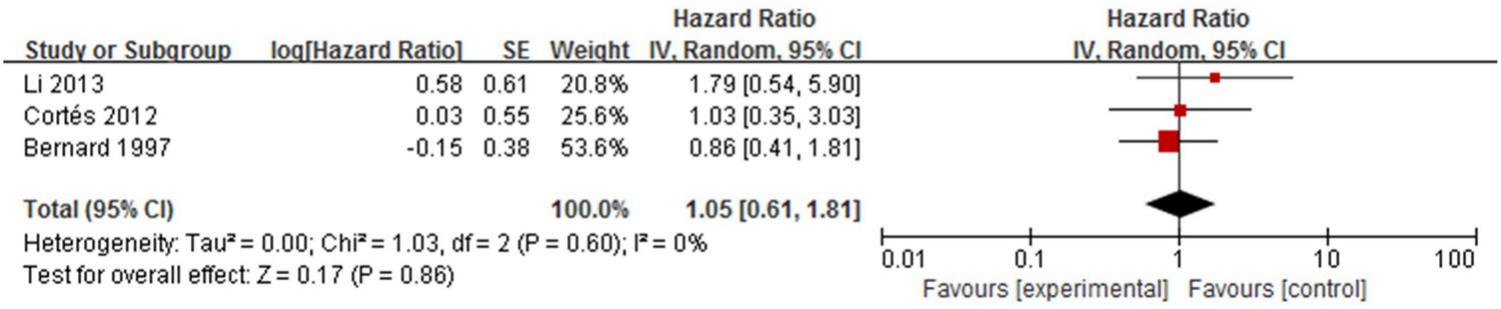
Forest plot of the association between positive IIF and bullous pemphigoid mortality.

The association between positive IIF and mortality in patients with bullous pemphigoid was analysed in three studies [9,18,19] (Fig 11). Positive IIF was defined as the presence of anti-basement membrane zone antibody, mainly IgG. Meta-analysis showed that the pooled HR was 1.05 (95%CI: 0.61–1.81, *P* = 0.86), and the results were not statistically significant. No heterogeneity was seen among the studies (*P* = 0.60, I^2^ = 0%).

#### 7. Dementia

**Fig 12:**
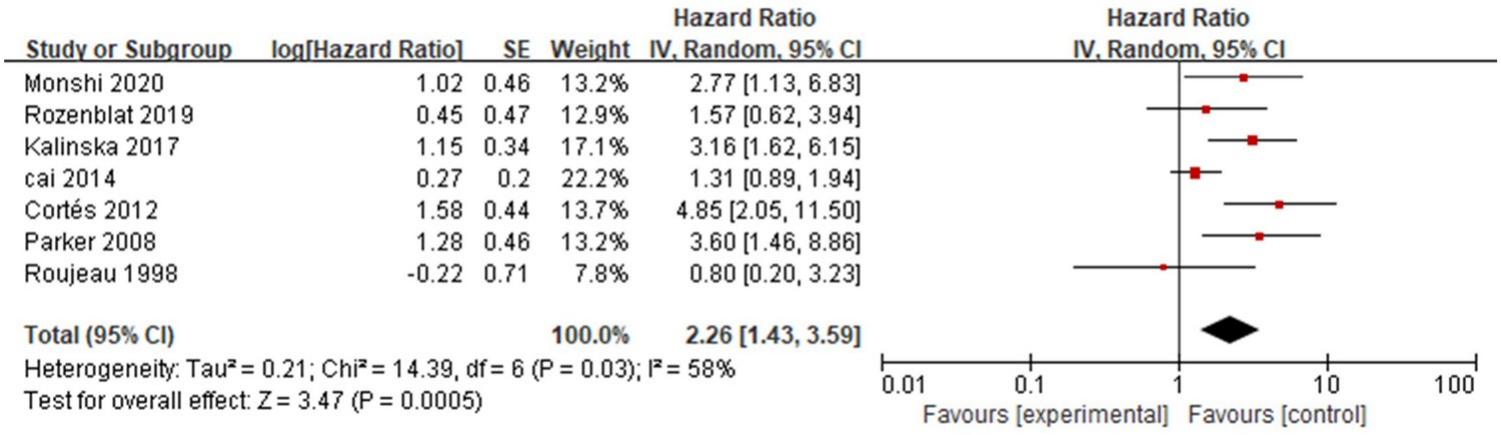
Forest plot of the association between dementia and bullous pemphigoid mortality.

Seven studies [10,11,13,16,19,21,23] analysed the association between dementia and mortality in patients with bullous pemphigoid (Fig 12). The overall pooled HR of dementia was 2.26 (95%CI: 1.43–3.59, *P*<0.001), and the results were statistically significant. The heterogeneity among the studies was moderate (*P* = 0.03, I^2^ = 58%).

The sensitivity analysis was performed on the studies. By removing each study one by one, it was found that unchanged effect of the studies (HR = 2.73, 95%CI: 1.79–4.15, *P*<0.001) and the heterogeneity was significantly reduced (P = 0.25, I^2^ = 24%) after removing the study of Cai [16], which might have been the source of heterogeneity among all studies. Further reading and evaluation of this study showed a large-sample size, clear outcome indicators, appropriate statistical methods and high quality score; therefore, this study could not be eliminated. The results showed statistical significance (HR = 2.47, 95%CI: 1.53~3.98, *P*<0.001) with similar heterogeneity (*P* = 0.03, I^2^ = 61%) after removing the study [23] that provided RR. The results showed an unchanged effect (HR = 2.75, 95%CI: 1.82–4.15, *P*<0.001) with no heterogeneity (P = 0.59, I^2^ = 0%) after excluding the studies [16,19,23] that the endpoint indicator was not one-year mortality, which indicating that assessing mortality in different way was the source of heterogeneity.

#### 8. Stroke

**Fig 13:**
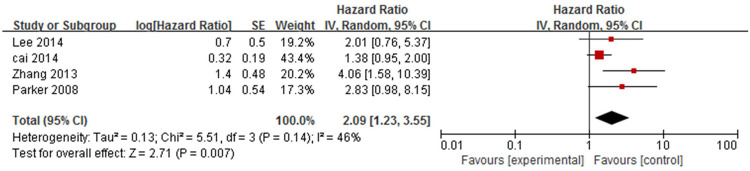
Forest plot of the association between stroke and bullous pemphigoid mortality.

Four studies [14,16,17,21] analysed the association between stroke and mortality in patients with bullous pemphigoid (Fig 13). The pooled HR was statistically significant (HR = 2.09, 95%CI: 1.23–3.55, *P* = 0.007). There was mild heterogeneity among the studies (*P* = 0.14, I^2^ = 46%).

#### 9. Hypertension

**Fig 14:**
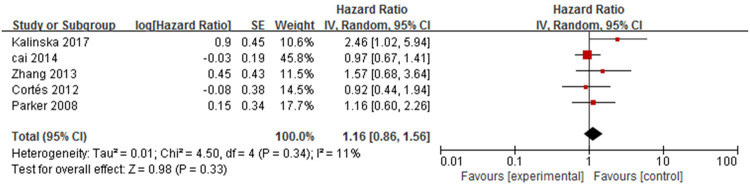
Forest plot of the association between hypertension and bullous pemphigoid mortality.

Five studies [13,16,17,19,21] analysed the association between hypertension and mortality in patients with bullous pemphigoid (Fig 14). The pooled HR of hypertension was not statistically significant (HR = 1.16, 95%CI: 0.86–1.56, *P* = 0.33). No heterogeneity was found among the studies (*P* = 0.34, I^2^ = 11%).

#### 10. Heart disease

**Fig 15:**
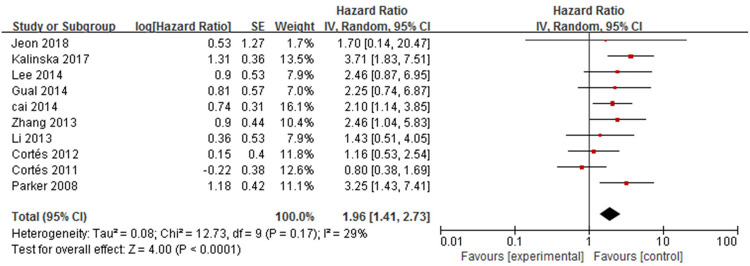
Forest plot of the association between heart disease and bullous pemphigoid mortality.

**Fig 16:**
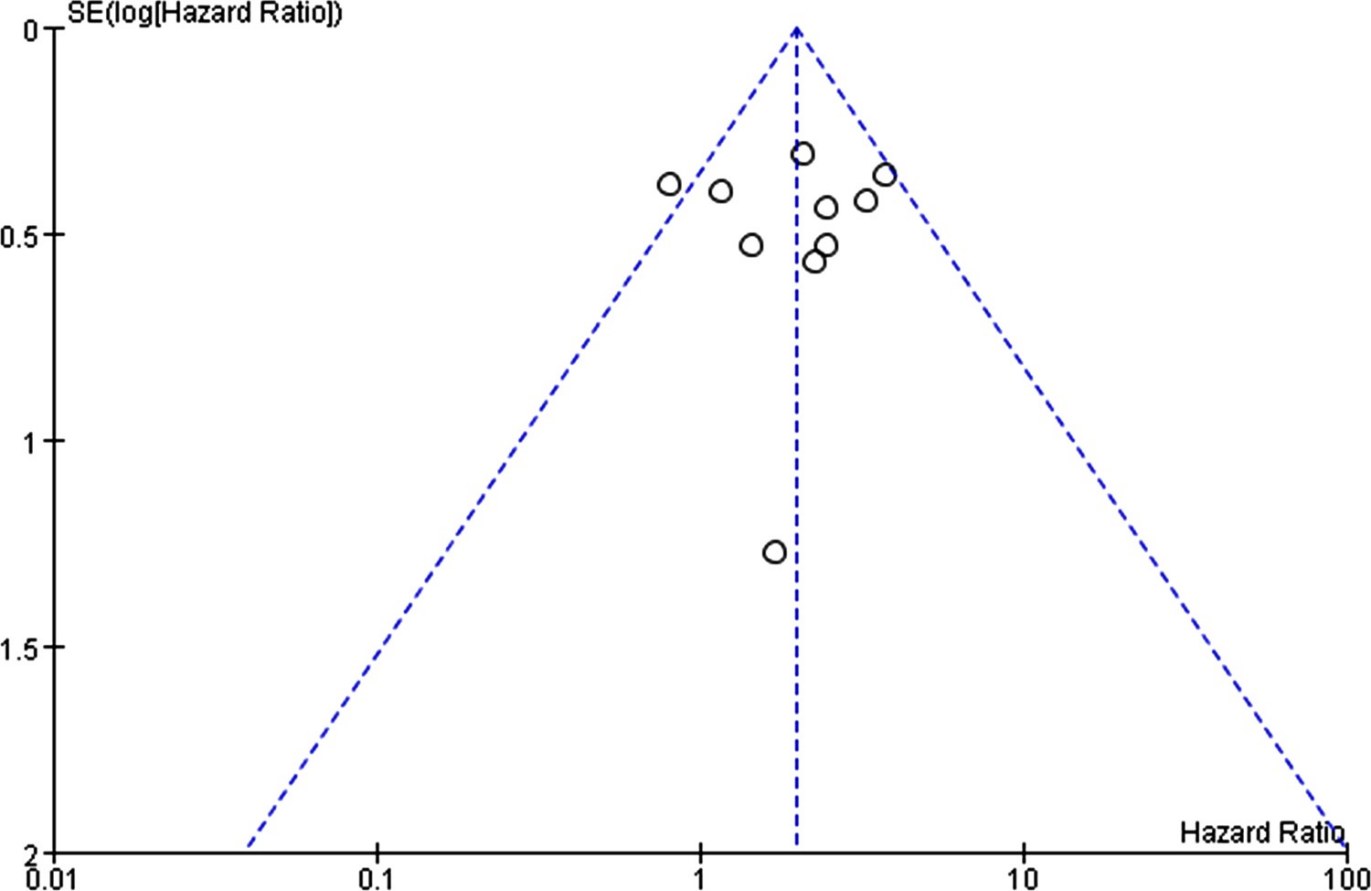
Funnel plot of the association between heart disease and bullous pemphigoid mortality.

**Fig 17:**
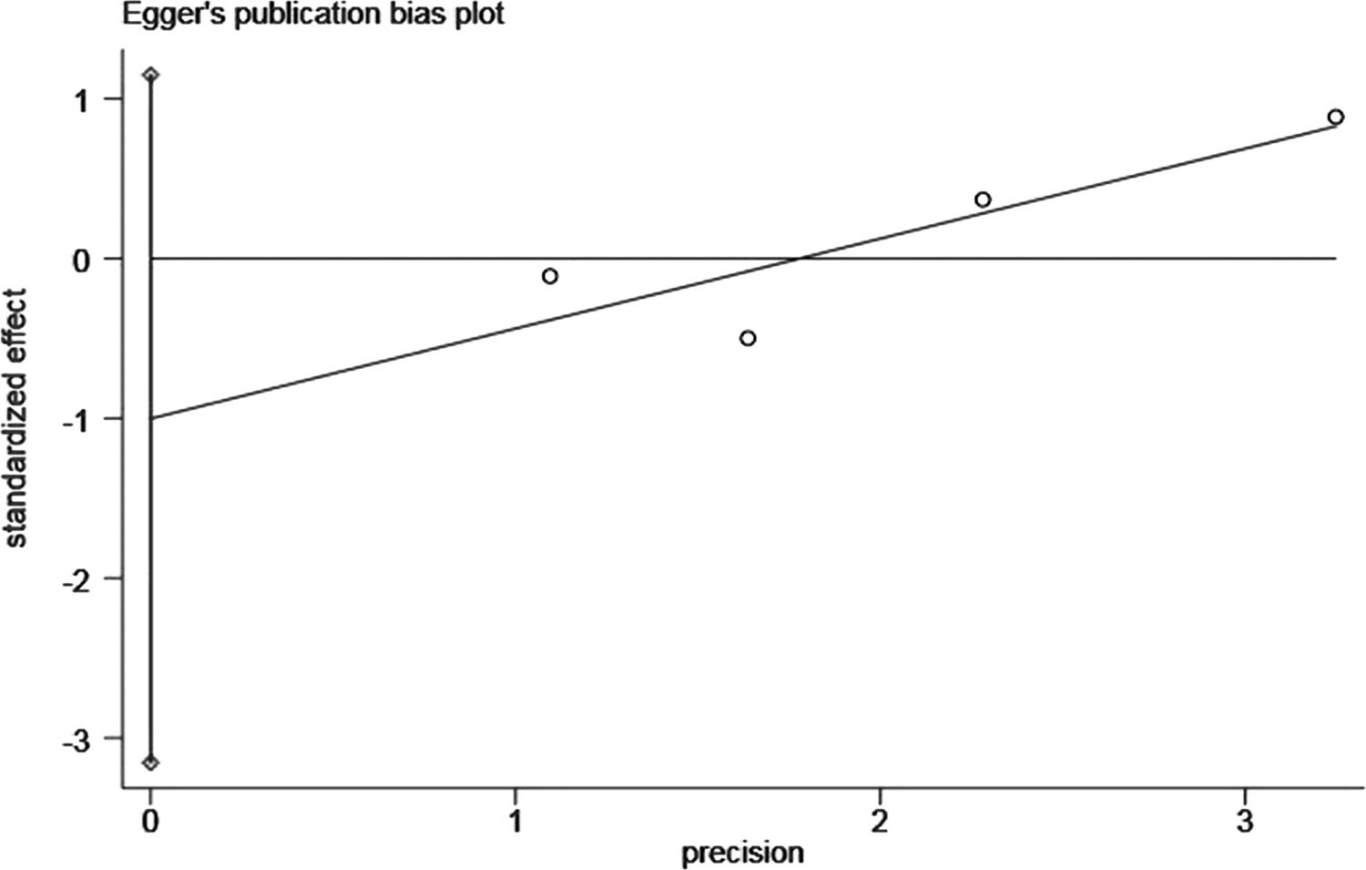
Egger’s publication bias plot of the association between heart disease and bullous pemphigoid mortality.

Ten studies [12–21] analysed the association between heart disease and mortality in patients with bullous pemphigoid (Fig 15). The overall pooled HR was 1.96 (95%CI: 1.41–2.73, *P*<0.001), and the results were statistically significant. Mild heterogeneity among the studies was found (*P* = 0.17, I^2^ = 29%). The funnel plot had symmetrical shape and was concentrated on the top of the graph (Fig 16). The non-significant Egger’s test results (*P*>0.05) (Fig 17) indicated the absence of publication bias.

#### 11. Diabetes mellitus

**Fig 18:**
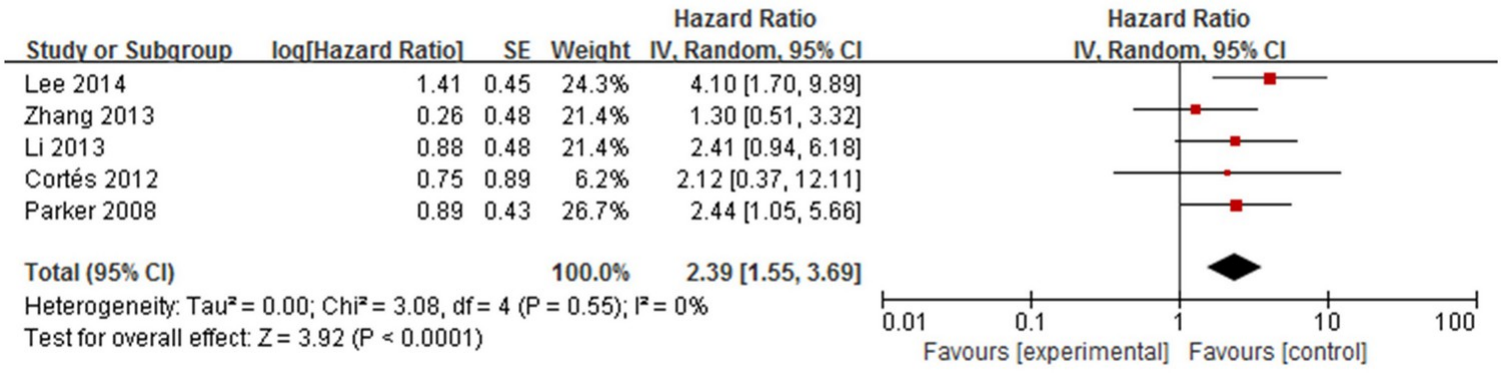
Forest plot of the association between diabetes mellitus and bullous pemphigoid mortality.

Five studies [14,17–19,21] analysed the association between diabetes mellitus and mortality in patients with bullous pemphigoid (Fig 18). Meta-analysis showed that the pooled HR was 2.39 (95%CI: 1.55–3.69, *P*<0.001), and the results were statistically significant. No heterogeneity was found among the studies (*P* = 0.55, I^2^ = 0%).

## Discussion

A total of 15 articles were included in this systematic review and meta-analysis. The 11 factors of age, sex, disease severity, general condition, bullous pemphigoid 180 antibody, IIF, dementia, stroke, hypertension, heart disease and diabetes, which may affect the prognosis of patients with bullous pemphigoid, were assessed by meta-analysis. The results showed that positive bullous pemphigoid 180 antibody, dementia, stroke, heart disease and diabetes were the risk factors for poor prognosis among patients with bullous pemphigoid.

Elderly patients are prone to develop bullous pemphigoid, which is often associated with various systemic diseases and has a poor prognosis because of the poor physical function and low immunity of this population. Current studies unanimously believed that old age at the time of diagnosis was one of the important factors affecting the prognosis of bullous pemphigoid. Liu’s meta-analysis confirmed this conclusion [29]. However, the results of our meta-analysis showed that there was a high degree of heterogeneity and publication bias among the studies. Subgroup and sensitivity analyses showed no heterogeneity source. And the absence of statistical significance after adding seven studies to adjust for publication bias suggested that publication bias may lead to overestimation of the impact of advanced age on the prognosis of patients with bullous pemphigoid. Therefore, it was unable to quantitatively evaluate the effect of advanced age on bullous pemphigoid mortality. In the future, more unified research data on age should be further strongly demonstrated.

Although only a few studies [18] found that sex affected bullous pemphigoid mortality, the results of this meta-analysis suggested that sex was not related to the prognosis of bullous pemphigoid. This was consistent with the results of most studies [17,19].

Roujeau [23] found that generalized disease was associated with increased risk of bullous pemphigoid mortality. Monshi [10] and Parker [21] showed that disease severity did not affect the prognosis of patients with bullous pemphigoid. However, the current meta-analysis showed high heterogeneity among the studies because of the different assessment methods of disease severity and the retrospective design of most studies. We did not perform quantitative evaluation of the effect of disease severity and bullous pemphigoid mortality to avoid the combined results bias that may have been resulted from the use of different evaluation methods for disease severity. Therefore, more prospective research data are needed in the future.

Cai [16] have defined poor general condition as a KPS of ≤40 (i.e., loss of autonomic function, long-term wheelchair or bed rest) and found that poor general condition increased the mortality of patients with bullous pemphigoid attributed to that long-term bed rest predisposed the patients to pneumonia and urinary tract infection. However, our meta-analysis had high heterogeneity and poor stability of the overall effect quantity, which may be related to the inconsistent evaluation criteria for the general condition among various studies. Therefore, it was unable to quantitatively evaluate the effect of the general condition on the mortality of bullous pemphigoid. In the future, a more uniform standard is needed to evaluate the general condition.

The serologic detection of bullous pemphigoid autoantibodies mainly relies on IIF and ELISA. IIF can detect the presence of IgG in the basement membrane zone, whereas ELISA can detect the existence of antibodies to bullous pemphigoid 180 and bullous pemphigoid 230 and monitor the serologic level of bullous pemphigoid 180 antibody [30]. This meta-analysis showed that the presence of the bullous pemphigoid 180 antibody was a risk factor for increased mortality in patients with bullous pemphigoid, whereas a positive IIF did not affect the prognosis of bullous pemphigoid. The antibody titer of bullous pemphigoid 180 may have reflected the severity of the disease, as well as the skin structural changes in patients with immune aging, old age, or poor general condition [10]. Therefore, the presence and high level of bullous pemphigoid 180 antibody indicated poor prognosis and should be paid attention to clinically.

Lai [31] has suggested an association between bullous pemphigoid and neurological diseases, including stroke, Parkinson’s disease, dementia, epilepsy, and multiple sclerosis. At present, the mechanism of the association between bullous pemphigoid and neurological diseases is based on the presence of the bullous pemphigoid auto-antigen in the brain of patients with neurological diseases. In patients with neurological diseases, the blood-brain barrier is seriously damaged and the auto-antigen may be released because of multiple minimally invasive injuries, microvascular lesions, or local inflammation; these can trigger an immune reaction and lead to the development of bullous pemphigoid [32,33]. More and more studies showed that the existence of neurological diseases affected the prognosis of patients with bullous pemphigoid [10,13]. This meta-analysis showed that among patients with bullous pemphigoid, the mortality rate increased nearly twice in the presence of dementia and stroke, which are the two most common types of neurological diseases, which was consistent with Liu’s study [29]. The mortality rate can be increased by neurological diseases itself and can further increase because of the autonomic dysfunction of patients with bullous pemphigoid; therefore, caution should be taken in patients with bullous pemphigoid and concomitant dementia and stroke.

Försti [34] found that cardiovascular disease was the most common comorbidity of bullous pemphigoid (about 76.3%). The existence of cardiovascular disease was reported to lead to poor prognosis among patients with bullous pemphigoid. Most studies defined coronary artery disease, cardiac arrhythmia, and heart failure as cardiovascular diseases [12,14,21], because these diseases were independent risk factors for death and the diuretics used could induce the progression and deterioration of patients with bullous pemphigoid [12]. This meta-analysis confirmed that the mortality of patients with bullous pemphigoid was increased by concomitant heart disease but not by concomitant hypertension, which was accidental. We speculated that the prognosis of patients with hypertension was relatively good after long-term drug control and monitoring. However, there was an interaction between heart disease and hypertension; therefore, more studies are needed to explore the different effects of heart disease and hypertension on the prognosis of bullous pemphigoid.

Diabetes mellitus is one of the common diseases in the elderly and is a usual comorbidity in patients with bullous pemphigoid. This meta-analysis showed that the mortality of patients with bullous pemphigoid and concomitant diabetes increased by two times. We speculated that patients with diabetes were prone to various infections, owing to the metabolic disorder, declining resistance and immunity and delayed wound healing in patients with bullous pemphigoid. Moreover, high blood glucose may lead to multi-systemic complications. Therefore, we should actively control blood glucose in patients with bullous pemphigoid and concomitant diabetes.

At present, many factors remain to be considered to be related with the prognosis of bullous pemphigoid. However, it was impossible to combine all these factors in this meta-analysis because of the lack of data. Mucosal involvement in patients with bullous pemphigoid was recently reported to have a relatively low incidence of 5.7% to 18.6% and was considered to be related to the severity of the disease [35]. Therefore, the presence of mucosal damage in patients with bullous pemphigoid disease may lead to a more serious disease with a poor prognosis. Some studies have analysed this viewpoint, but the results were not statistically significant [16,18]. Low serum albumin levels and high erythrocyte sedimentation rate could reflect disease severity and have been considered to increase the mortality of patients with bullous pemphigoid. However, these are non-specific indicators that can be easily affected by several factors, such as other acute and chronic illnesses; therefore, they could not be evaluated as a single indicator. Concomitant kidney disease and malignant tumours were considered to be poor prognostic factors and may inevitably increase the mortality of patients with bullous pemphigoid, owing to the high mortality of each disease. However, this could not be confirmed by sufficient research and would need further exploration. Glucocorticoid is the first-line treatment of bullous pemphigoid. Compared with systemic glucocorticoids, topical glucocorticoids have been reported to reduce mortality [36,37]. Although there was a lack of data in this study, application of topical glucocorticoids to the whole body for generalized bullous pemphigoid might lead to skin atrophy, deterioration, and other side effects. In addition, compared with high-dose glucocorticoid alone, the combination of glucocorticoids and immunosuppressive agents or antibiotics has been considered to reduce mortality [16,38] because of the synergistic effect of combined therapy and the reduction of adverse reactions caused by glucocorticoids. However, the research data were few, and the retrospective design of studies precluded control of the treatment scheme, administration route and dosage according to the disease situation. Therefore, more prospective large-sample studies are needed in the future to further demonstrate the impact of a treatment scheme on the prognosis of bullous pemphigoid. In the recent two years, the effect of statins as a new indicator has emerged and had been considered to reduce the mortality of patients with bullous pemphigoid [11]. The specific mechanism may be immunomodulatory by reduction of the metabolites of the L-mevalonate pathway and up-regulation of the immune response through inhibition of 3-hydroxy-3-methylglutaryl CoA reductase [39]. Attention should be paid to the possible protective role of statins for the clinical prevention and treatment of patients with bullous pemphigoid. Hospitalisation days have been considered to be associated with increased mortality in patients with bullous pemphigoid. Monshi [10] believed that longer hospitalization days reflected the degree of disease, the complications in the disease course, and the occurrence of adverse events during treatment. However, there had been no uniform standard for the length of hospitalization, and this index can be easily affected by many factors, such as medical conditions and treatment levels.

### Limitations

There were some limitations in evaluating the results of this meta-analysis. First, most of the studies included were retrospective, which may have led to bias in data collection and selection. Second, differences in treatment modalities among the studies might have affected overall mortality. Third, the difference in follow-up time among the studies might have affected the comparability of the research results. Last, some factors were analysed in only one or two studies and cannot be quantitatively evaluated; this may have affected the comprehensiveness of the research results. Therefore, more prospective and large-sample studies are needed to improve and update the prognostic factors for mortality in bullous pemphigoid.

## Conclusion

This systematic review and meta-analysis demonstrated that advanced age, positive bullous pemphigoid 180 antibody, poor general condition, concomitant dementia, stroke, heart disease, and diabetes mellitus were the factors that can influence the poor prognosis for mortality in patients with bullous pemphigoid. Although there were some limitations in this study, the results were obtained by summarising the data of multivariable analyses, and the conclusion was relatively reliable. Therefore, it may have clinical value for the prognosis and prevention of bullous pemphigoid.

## Supporting information

S1 Checklist (DOC)
